# Brittleness mechanisms and toughening strategies of electrical insulating polymer materials under extreme cryogenic conditions

**DOI:** 10.1039/d6ra02004f

**Published:** 2026-07-02

**Authors:** Yuhan Deng, Mengjin Li, Yuhong Yuan, Baowen Xing, Wanchuan Liu, Jingrui Liu

**Affiliations:** a College of Materials Science and Engineering, Chongqing University Chongqing 400045 China; b School of Physics, Chongqing University Chongqing China; c College of Electrical Engineering, Chongqing University Chongqing 400044 China; d School of Mechanical Engineering, Tianjin University of Technology Tianjin 300384 China; e Department of Chemistry, College of Literature, Science, and the Arts, University of Michigan Ann Arbor MI 48109 USA; f College of Engineering and Applied Science, University of Cincinnati Cincinnati OH 45221 USA liu3jr@mail.uc.edu; g Chongqing University-University of Cincinnati Joint Co-op Institute, Chongqing University Chongqing 400044 China

## Abstract

Extreme cryogenic environments in superconducting and liquid hydrogen systems pose severe challenges to electrical insulation, where polymer embrittlement leads to catastrophic failure. This review systematically elucidates the multiscale mechanisms of low-temperature embrittlement and evaluates state-of-the-art toughening strategies. We analyze the intrinsic nature of brittleness by correlating macroscopic failure with molecular-scale phenomena, including chain segment freezing and the suppression of secondary relaxations. Mainstream toughening approaches—ranging from intrinsic molecular architecture design to extrinsic nano-reinforcement—are critically discussed and compared based on their efficacy in cryogenic toughness enhancement. Distinct from existing literature, this work synthesizes the synergy between insulation performance and mechanical toughness. Finally, we propose concrete research directions, focusing on multiscale simulation-guided design and intelligent materials for multi-field coupled environments.

## Abstract 2

The review reveals how chain freezing, free-volume collapse and secondary relaxation suppression induce dielectric embrittlement at cryotemperatures, and compares intrinsic/extrinsic toughening for design for superconducting & hydrogen systems.

## Graphical abstract

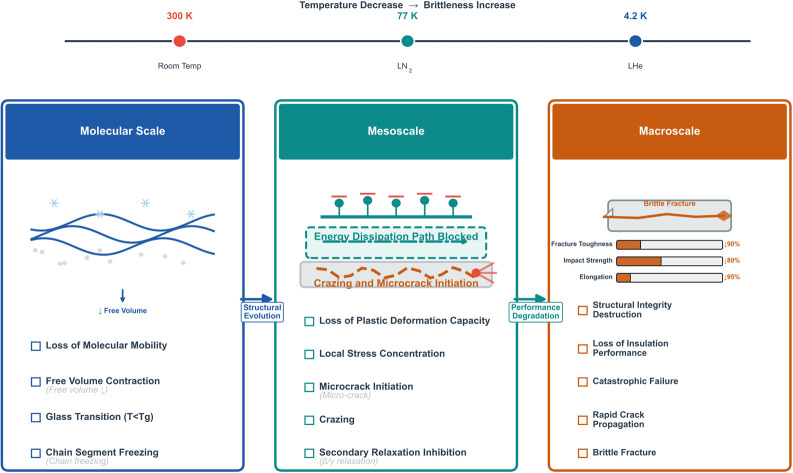

## 1. Introduction

Electrical insulating polymers constitute the material foundation of insulation systems in power apparatus and electronic devices, and therefore largely determine the reliability, compactness, and service lifetime of such equipment. Traditionally, insulating polymers have been primarily employed under ambient or elevated temperature conditions. However, driven by advances in energy, aerospace, scientific research, and medical technologies, an increasing number of applications—such as superconducting power devices, liquid hydrogen/liquid nitrogen storage and transportation systems, deep-space probes, and large-scale particle accelerators—are extending electrical engineering applications into extreme cryogenic regimes, typically ranging from the liquid nitrogen temperature region (77 K) to the liquid helium temperature region (4.2 K).^1,2^

Under extreme cryogenic conditions, most polymer materials undergo a transition from ductile to brittle behavior. Macroscopically, this transition is characterized by a sharp reduction in fracture toughness and impact strength.^3^ Even minor stress concentrations or thermo-mechanical stresses can trigger the initiation and rapid propagation of microcracks within the material,^4^ ultimately leading to brittle fracture of the insulation structure.^5^ Such failure not only degrades electrical insulation performance but may also compromise mechanical integrity, potentially resulting in catastrophic consequences. For example, microcracks induced by cryogenic embrittlement in superconducting magnet insulation systems may propagate under high electromagnetic stresses, causing insulation breakdown and magnet quenching.^4^ Similarly, thermal cycling-induced cracking in low-temperature electronic packaging of spacecraft can directly lead to functional failure.^3^

Although there have been numerous studies on the cryogenic mechanical properties of polymers, most works remain focused on single-scale descriptions or empirical modifications of specific materials. There is still a critical lack of systematic synthesis ranging from molecular chain dynamics to macroscopic fracture mechanics, especially considering the unique operating environments of power equipment (*e.g.*, coupling of strong electric fields and thermal stresses). In particular, there is a lack of a unified theoretical framework and effective cross-scale design criteria for balancing dielectric strength and mechanical toughness at extreme cryogenic temperatures. This research gap significantly limits the R&D efficiency of high-performance cryogenic insulation materials and their application in next-generation superconducting power systems.

Therefore, investigating the physical mechanisms of cryogenic embrittlement in insulating polymers and developing effective toughening strategies have become urgent research priorities at the intersection of cryogenic electrical engineering and polymer materials science. In order for readers to systematically master this complex subject, this paper constructs a logical framework from mechanism analysis to modification practices and finally to future trends: first, taking typical epoxy-based insulating polymers as the primary object and starting from theories such as molecular motion and free volume, the fundamental nature and influencing factors of cryogenic embrittlement are discussed in depth to lay a theoretical foundation for understanding the root causes of failure; next, based on the aforementioned mechanisms, representative intrinsic and extrinsic toughening strategies are summarized, with a comparative analysis of the suitability of different strategies in extreme cryogenic environments; finally, current technical bottlenecks are combined to present challenges and future perspectives. The goal is to provide theoretical guidance for failure prediction and material optimization in cryogenic electrical equipment, such as superconducting magnets and liquid hydrogen storage systems, thereby supporting the engineering development of highly reliable insulation systems.^5^

## 2. Multiscale mechanisms of cryogenic embrittlement

### 2.1 Freezing of chain segmental motion and glass transition

The mechanical toughness of polymers is fundamentally governed by the cooperative mobility of molecular chains and hierarchical chain segments, ranging from side groups to backbone segments.^6^ Such molecular motions are highly sensitive to temperature, and the glass transition represents the key thermophysical process regulating this behavior. When the temperature is above the glass transition temperature (*T*_g_), secondary intermolecular interactions—such as van der Waals forces and hydrogen bonding—are relatively weak, allowing chain segments to obtain sufficient thermal activation energy to undergo rotational motion around the backbone, inter-chain slippage, and entanglement rearrangement. Under these conditions, the material remains in a rubbery state.^7^ Upon external loading, irreversible segmental motions effectively dissipate energy and alleviate stress concentration, thereby preventing brittle fracture and enabling good impact resistance and ductility at the macroscopic scale.^8^

When the temperature decreases below *T*_g_, the available thermal activation energy becomes insufficient to overcome the energy barriers for segmental motion. As a result, chain mobility is significantly “frozen,” leaving only small elastic deformations associated with bond length and bond angle variations (*i.e.*, general elastic deformation). The material rapidly transitions into a glassy state, and its mechanical response shifts from plastic to brittle behavior. Molecular dynamics simulations indicate that the freezing of segmental motion under cryogenic conditions exhibits scale dependence, with short side-chain segments typically freezing at lower temperatures than backbone segments. Under extreme cryogenic conditions, such as the liquid nitrogen temperature region (77 K, approximately −196 °C), the motion of chain segments at all hierarchical levels is nearly completely suppressed. Internal stresses cannot be released through segmental rearrangement, and even minor external loading may trigger instantaneous fracture.^9^

In electrical insulation applications, commonly used polymers exhibit glass transition temperatures that differ markedly from extreme cryogenic temperatures, which is a key factor contributing to embrittlement. Bisphenol-A-based epoxy resins, widely used as insulating matrix materials, typically possess *T*_g_ values of approximately 110–140 °C,^10^ while phenolic epoxy resins exhibit *T*_g_ values in the range of 120–150 °C. These rigid systems therefore operate far below their *T*_g_ under cryogenic conditions. Under cryogenic conditions, dense molecular packing severely restricts segmental rotation and slippage, causing the impact strength to decrease from approximately 20–30 kJ m^−2^ at room temperature to about 2–5 kJ m^−2^ at 77 K, indicating pronounced embrittlement.^11^ Even flexible electrical insulating polymers with relatively low *T*_g_ experience substantial and often irreversible loss of toughness when exposed to temperatures far below their *T*_g_,^12^ as summarized in Table 1.

**Table 1:** Cryogenic properties of typical electrical insulating polymers^a^

Polymer type	Chemical structure	*T*_g_ (°C)	*K*_IC_ at 77 K (MPa m^1/2^)	Main applications	Cryogenic embrittlement
Bisphenol A epoxy	Rigid benzene ring + ether bond	120–150	0.6–1.0	Superconducting magnet insulation	Severe embrittlement, impact strength typically decreases from ∼20–30 kJ m^−2^ (RT) to ∼2–5 kJ m^−2^ (77 K)
Cycloaliphatic epoxy	Saturated alicyclic structure	140–180	0.8–1.5	Aerospace encapsulation	Significant embrittlement
Polyurethane (TPU)	Soft segment + hard segment block	−50 to −20	2.0–4.0	Cryogenic cables, foam insulation	Modulus decreases but retains certain toughness
Polyimide (PI)	Imide ring structure	300–400	1.65–2.8	Flexible circuits, film insulation	Relatively brittle, but superior to epoxy
Polytetrafluoroethylene (PTFE)	Fluorocarbon chain	−20 to −120	3.0–5.0	Seals, bearings	Cold flow, strain reduced by 99% at 77 K
Polyetheretherketone (PEEK)	Aromatic ether ketone	143	4.0–6.0	Connectors, structural components	Relatively best, highest strength at 77 K
Bismaleimide (BMI)	Imide + aromatic ring	210–230	1.5–2.5	High-temperature insulation	Moderate embrittlement
Low-density polyethylene (LDPE)	Linear polyethylene chain	−110	2.5–4.0	Cryogenic coating	Increased crystallinity, reduced toughness

a The *T*_g_ data are sourced from the Polymer Handbook and material databases. The *K*_IC_ fracture toughness data at 77 K represent an estimated typical range due to significant variations among resin systems and limited literature; specific values should be determined based on the actual resin formulation and testing conditions. The *K*_IC_ data for Kapton and LARC-TPI are derived from ref. 13, while the pure epoxy impact strength data are cited from ref. 14.

Low-density polyethylene (LDPE, *T*_g_ ≈ −125 °C), ethylene-vinyl acetate copolymer (EVA, *T*_g_ ≈ −40 °C), and polytetrafluoroethylene (PTFE, *T*_g_ ≈ −120 °C) are representative flexible polymers that are widely used as insulation coating materials in cryogenic electrical equipment. Studies have shown that, although the *T*_g_ of LDPE and PTFE are higher than 77 K, their crystallinity increases at cryogenic temperatures, and crystalline confinement further restricts chain motion in amorphous regions: the entangled linear chains of LDPE are completely locked, leading to a significant drop in toughness; PTFE with an intrinsically rigid fluorocarbon backbone has nearly fully frozen chain motion and markedly inhibited cold flow, with its strain at 77 K reduced by 99% from room temperature. EVA has a higher *T*_g_ and enters a deep glassy state at 77 K, where both main chain segmental motion and side group secondary relaxation are strongly suppressed, resulting in complete loss of synergistic deformability.^15^ In addition, the freezing of molecular chain segmental motion under cryogenic conditions promotes the accumulation of internal residual stresses, thereby increasing the risk of brittle fracture. This effect is particularly pronounced in thick-walled electrical insulation components, where stress relaxation is more limited under cryogenic conditions.

### 2.2 Free volume theory and its evolution at cryogenic temperatures

Free volume theory provides a classical framework for interpreting the nature of the glass transition and the low-temperature mechanical behavior of polymers. By revealing the intrinsic relationship between microscopic voids and molecular chain mobility, it offers important theoretical support for understanding the embrittlement mechanisms of electrical insulating polymers under extreme cryogenic conditions. Free volume refers to the three-dimensional microscopic voids within a polymer that are not occupied by molecular backbones or side groups. It includes intrinsic free volume arising from packing defects of molecular chains and excess free volume generated by thermal motion. These two components collectively provide the necessary spatial allowance for chain segment rotation, slippage, and rearrangement, and their content and distribution directly determine the activation threshold of segmental motion. The temperature-dependent evolution of free volume can be quantitatively characterized by positron annihilation lifetime spectroscopy (PALS), with supplementary validation from pressure–volume–temperature (PVT) testing and dynamic mechanical analysis (DMA).

The amount of free volume varies systematically with temperature and is closely coupled with phase transitions in polymers. When the temperature is above *T*_g_, intense thermal motion of molecular chains leads to a continuous increase in excess free volume. With the spatial buffering effect provided by free volume, chain segments can overcome intermolecular interactions and undergo cooperative motion and entanglement reconstruction. Under such conditions, mechanical energy can be dissipated through plastic deformation mechanisms, including shear yielding and crazing, thereby maintaining material toughness. When the temperature decreases below *T*_g_, the thermal motion of molecular chains is significantly reduced, and the excess free volume gradually contracts and stabilizes.^16^ Only a small fraction of intrinsic free volume remains, and its spatial distribution becomes less uniform, forming isolated clusters of microvoids.

Under extreme cryogenic conditions (77 K and below), the free volume is further compressed to a minimal level. The connectivity between adjacent void clusters disappears, and the spatial conditions required for segmental motion are severely restricted, preventing effective molecular displacement under external loading. This extreme contraction not only increases the elastic modulus and macroscopic hardness of the material but also suppresses the activation of plastic deformation mechanisms, eliminating effective energy dissipation pathways. Electrical insulating polymers such as epoxy resins, polyethylene, and polyimides exhibit intensified molecular packing and strengthened intermolecular interactions at cryogenic temperatures, forming a rigid network-like structure.^17^

When external stress is applied, the scarcity of free volume prevents stress relaxation through segmental rearrangement, causing rapid energy concentration at internal defects (*e.g.*, microvoids, impurity interfaces, and chain ends) and crack tips. Finite-element and micromechanical studies have shown that thermal stresses induced by CTE mismatch in cryogenic polymer composite systems may reach several tens to more than 100 MPa depending on cooling rate, geometry, and interfacial constraint conditions. For brittle epoxy systems operating at 77 K, the tensile fracture strength and local failure stress are often within a comparable range, especially near interfacial defects and crack tips where stress concentration occurs. Under these conditions, the local stress intensity may exceed the fracture threshold and trigger unstable crack propagation. Under such conditions, cracks cannot be blunted through shear yielding and instead propagate rapidly through the matrix, resulting in typical brittle fracture without noticeable plastic deformation.^18^ As summarized in Table 1, this behavior is consistent with the significant reduction in impact strength and fracture toughness—for instance, from approximately 20–30 kJ m^−2^ at room temperature to about 2–5 kJ m^−2^ at 77 K for bisphenol A epoxy—observed in insulating polymers at extreme cryogenic temperatures. In addition, the evolution of free volume under cryogenic conditions also influences dielectric properties, where free volume contraction and void isolation alter carrier transport pathways^19^ and induce local electric field distortion, raising dielectric loss and breakdown risk,^20^ producing a coupled effect with embrittlement behavior and further degrading the service reliability of insulating materials.

### 2.3 Suppression of secondary relaxation processes

Although free-volume contraction and secondary-relaxation suppression are discussed separately for clarity, both processes originate from the same reduction in thermal activation energy and evolve cooperatively at cryogenic temperatures. The contraction and isolation of free volume further restrict localized molecular motions responsible for β- and γ-relaxations, while the loss of these relaxations reduces the dynamic redistribution capability of free volume.

After polymers enter the glassy state, although large-scale backbone segmental motion becomes frozen, small-scale molecular motions still exist. These motions are dominated by localized units such as side groups, short backbone sequences, branched structures, and torsional motions of functional groups, collectively referred to as secondary relaxation processes. According to their relaxation temperatures, these processes are typically classified as β and γ relaxations. Secondary relaxations constitute the primary microscopic energy dissipation mechanisms in glassy polymers and play an important role in regulating the low-temperature toughness of electrical insulating polymers. Compared with backbone segmental motion, secondary relaxations require lower activation energy and smaller spatial freedom, allowing them to remain partially active in temperature ranges slightly below *T*_g_, thereby acting as an important buffer against brittle fracture.

To determine when secondary relaxation suppression becomes more influential than cooperative chain segment freezing in governing cryogenic embrittlement, the temperature dependence of relaxation dynamics must be considered. The α-relaxation, associated with cooperative chain segmental motion, follows Vogel–Fulcher–Tammann (VFT) behavior and becomes dynamically arrested near *T*_g_ on experimental timescales. In contrast, β-relaxation, which originates from localized molecular motions, generally follows Arrhenius behavior with lower activation energy. Consequently, β-relaxation persists to lower temperatures than α-relaxation but progressively falls outside experimentally accessible timescales below the characteristic β-relaxation temperature (typically ∼200–250 K in epoxy systems). At 77 K, both α- and β-motions are effectively frozen, eliminating most molecular-scale energy dissipation pathways. Within the intermediate regime (*T*_β_ < *T* < *T*_g_), the two mechanisms remain coupled: suppression of cooperative chain motion limits large-scale plasticity, while residual β-relaxation can still contribute to crack-tip energy dissipation. At deeper cryogenic temperatures, secondary relaxation suppression becomes increasingly dominant because localized molecular mobility is also fully immobilized.

Different types of secondary relaxation contribute differently to low-temperature mechanical behavior. β-Relaxation generally originates from localized torsional motion of short backbone segments or cooperative motion between side groups and the backbone, and its activity directly affects the impact toughness and low-temperature plasticity of glassy polymers. γ relaxation is mainly associated with independent rotational motion of side groups such as methyl or ethyl groups. Although its energy dissipation efficiency is lower than that of β-relaxation, it can still provide limited plastic deformation capacity at lower temperatures. These relaxation processes act as microscopic energy dissipation sites that absorb part of the applied mechanical energy through localized motion, thereby delaying crack initiation and propagation and mitigating cryogenic embrittlement.

The activity of secondary relaxation is highly temperature-dependent. When the temperature decreases to 77 K or below, most secondary relaxation processes are strongly suppressed or completely frozen due to insufficient activation energy. Molecular dynamics studies indicate that the amplitude of thermal vibration of relaxation units decreases significantly under extreme cryogenic conditions and becomes insufficient to overcome intermolecular potential barriers.^21^ For representative electrical insulating polymers, the β-relaxation occurs at −50 to 0 °C (bisphenol A epoxy),^22^ −25 to 0 °C (LDPE),^23^ 20 to 30 °C (PTFE),^24^ and 50 to 100 °C (PI),^25^ while the γ-relaxation locates at −100 to −70 °C (epoxy), −123 to −110 °C (LDPE),^23^ −125 to −75 °C (PTFE),^24^ and −100 to −75 °C (PI).^25^ When the temperature decreases to 77 K or below, β-relaxation is fully frozen in all above polymers, and only LDPE and PTFE retain extremely weak γ-relaxation activity. Consequently, the intensity of β and γ relaxation peaks decreases sharply, and the material loses its remaining microscopic energy dissipation pathways.

Once secondary relaxation is fully suppressed, external stress applied to electrical insulating polymers under cryogenic conditions cannot be dispersed through any microscopic molecular motion. Instead, stress rapidly concentrates at internal defects (*e.g.*, chain imperfections and interfacial voids) and crack tips, ultimately leading to brittle fracture without plastic deformation.^26^ The resulting embrittlement is more severe than that caused solely by backbone segment freezing. Therefore, the type, activity, and cryogenic stability of secondary relaxation processes are key factors determining the low-temperature toughness of polymers. Studies have shown that polymers exhibiting abundant and cryogenically active β-relaxation typically demonstrate superior low-temperature toughness.^27^ To this end, molecular design strategies for regulating secondary relaxation mainly include introducing flexible segments to construct cryogenically stable β-relaxation, regulating crosslink density to balance network rigidity and segmental mobility, and designing branched and hyperbranched structures to increase localized motion units, which will be explained in detail in Section 3.1. It should be noted that the suppression of global segmental mobility at cryogenic temperatures does not completely eliminate all localized deformation processes. Under highly concentrated stress fields, such as near crack tips, interfaces, or cavitated particles, confined nanoscale molecular rearrangements may still occur and contribute to limited energy dissipation. Different types of secondary relaxation arise from distinct chemical moieties. In epoxy networks, β-relaxation is primarily attributed to localized torsional motion of hydroxyether segments and rotational fluctuations of phenyl rings (activation energy 40–70 kJ mol^−1^), while γ-relaxation originates from methyl group rotations and small-angle motions of unreacted chain ends (15–30 kJ mol^−1^). These motions have different temperature dependencies: phenyl ring flips persist to approximately 150–200 K, whereas ether bond rotations become frozen above 200–250 K. Importantly, DMA studies have shown a positive correlation between β-relaxation intensity (tan *δ* peak height) and cryogenic fracture toughness. The positive correlation between β-relaxation intensity and cryogenic fracture toughness is supported by quantitative evidence from multiple experimental studies. Sawa *et al.* simultaneously measured relaxation behavior and cryogenic *K*_IC_ across epoxy systems with systematically varied plasticizer content and crosslink density, and explicitly concluded that *K*_IC_ at cryogenic temperatures bears a close quantitative relation to the degree of residual molecular relaxation activity; the benefit of plasticizers diminished progressively toward liquid helium temperature, confirming that molecular relaxation—rather than bulk compliance—governs low-temperature fracture toughness.^28^ Hartwig further demonstrated, through systematic compilation of sub-*T*_g_ DMA damping spectra across multiple polymer classes, that the tan *δ* peak intensity of the β-relaxation is directly associated with crack-tip energy dissipation capacity and crack-propagation resistance in the liquid-nitrogen temperature range: polymers exhibiting more pronounced DMA damping peaks consistently showed higher fracture toughness at cryogenic temperatures, because the measurement of *K*_IC_ inherently involves adiabatic heating and localized plastification at the crack tip, the extent of which depends on the residual sub-*T*_g_ relaxation properties of the material.^29^ These principles are quantitatively corroborated within individual studies: Gaarud *et al.* characterized multiple epoxy formulations by DMA and identified β-relaxation peaks of differing intensities (local tan *δ* maxima at approximately −80 to −100 °C), while simultaneously measuring *K*_IC_ at 77 K; the rigid CTD101K system, which exhibited the weakest β-relaxation signal, achieved a ∼60% increase in 77 K *K*_IC_ upon introduction of flexibiliser DY040, a modification that measurably enhanced the secondary relaxation peak.^30^ Studer *et al.* further confirmed this trend: enhancing sub-*T*_g_ β-relaxation intensity through flexible aliphatic amine chain extenders raised *K*_IC_ at 77 K from 2.0 to 5.3 MPa m^1/2^ (+165%), as already summarized in Tables 2 and 3 of this paper. Collectively, these results demonstrate a consistent positive correlation between DMA β-relaxation peak intensity and *K*_IC_ at 77 K across representative epoxy systems.

**Table 2:** Comparison of toughening strategies for cryogenic applications

Toughening strategy	Toughening mechanism	Cryogenic effectiveness (77 K)	Tested property	Resin system	Impact on electrical properties	Process complexity	Cost	Typical application
Flexibilizer (2-heptylamine)	Increased molecular mobility, reduced crosslink density	*K*_IC_ increased by ∼165% (from 2.0 to 5.3 MPa m^1/2^)^1^	Fracture toughness *K*_IC_	DGEBA + MPD	*T*_g_ decreased to 50–100 °C	Low	Medium	Superconducting magnet insulation
Rubber particle toughening (NR 15 phr)	Induce highly localized shear banding and stress-assisted deformation near cavitated particles	*K*_IC_ increased by 48.3% (from 1.64 to 2.43 MPa m^1/2^); tensile strength increased by 40.2% (ref. 43)	Fracture toughness *K*_IC_ + tensile strength	DGEBF + DETD	Increased dielectric constant, decreased *T*_g_	Medium	Medium	Structural adhesives, composites
Thermoplastic toughening (PES 10 phr)	Phase separation energy dissipation, PES plastic deformation, crack deflection	*K*_IC_ increased by 48% (from 1.727 to 2.551 MPa m^1/2^); *G*_IC_ increased by 180% (ref. 44)	Fracture toughness *K*_IC_ + strain energy release rate *G*_IC_	Epoxy + anhydride	*T*_g_ unchanged or slightly increased	Medium	High	Aerospace composites, cryogenic storage tanks
Nano-SiO_2_ toughening (5–20 wt%)	Crack deflection + plastic deformation	Improvement observed (magnitude varies with content)^45^	Tensile strength/flexural strength	DGEBA + anhydride	Potential increase in dielectric strength	High	Medium	Electronic encapsulation
CNT toughening (0.5 wt%)	Bridging + pull-out + thermal conduction	Impact strength 35.56 kJ m^−2^ (absolute value)^46^	Impact strength	DGEBF + DETD	Electrical conductivity risk	High	High	Multifunctional structures
Hyperbranched polymer (H30 10 wt%)	Intramolecular free volume	Impact strength increased by 26.3%; tensile strength increased by 17.7% (ref. 47)	Impact strength + tensile strength	DGEBA + DETD + MeTHPA	*T*_g_ unchanged	Medium	High	High-end encapsulation
Hyperbranched polymer (HPB 10 wt%)	Intramolecular free volume	Impact strength increased by 59.4% (ref. 17)	Impact strength	DGEBA + MNA	Minor impact	Medium	High	High-end encapsulation
Hyperbranched polymer grafted boron nitride (HBP-BN 4 wt%)	Intramolecular free volume + BN crack deflection/bridging	Impact strength increased by 135%; tensile strength increased by 72% (ref. 48)	Impact strength + tensile strength	DGEBA + MNA	Insulation maintained	Medium	High	High-end encapsulation

**Table 3:** Comparative cryogenic toughening performance of modified epoxy systems at 77 K based on literature-reported metrics^a^

Toughening strategy	Resin system	Tested property	Performance data at 77 K	Change relative to neat epoxy
Neat epoxy resin	DGEBF + DETD	Impact strength	24.0 ± 2.3 kJ m^−2^ (ref. 31)	—
Neat epoxy resin	DGEBF + DETD	Fracture toughness *K*_IC_	1.64 MPa m^1/2^ (ref. 43)	—
Neat epoxy resin	DGEBA + MPD	Fracture toughness *K*_IC_	2.0 MPa m^1/2^ (ref. 1)	—
Flexibilizer (2-heptylamine)	DGEBA + MPD	Fracture toughness *K*_IC_	5.3 MPa m^1/2^	Increased by ∼165% (from 2.0)^1^
Rubber particle toughening (NR 15 phr)	DGEBF + DETD	Fracture toughness *K*_IC_	2.43 MPa m^1/2^	Increased by 48.3% (from 1.64)^43^
Rubber particle toughening (NR 15 phr)	DGEBF + DETD	Tensile strength	Not quantitatively reported in the original study	Increased by 40.2% (ref. 43)
Thermoplastic toughening (PES 10 phr)	Epoxy + anhydride	Fracture toughness *K*_IC_	2.551 MPa m^1/2^	Increased by 48% (from 1.727)^44^
Thermoplastic toughening (PES 10 phr)	Epoxy + anhydride	Strain energy release rate *G*_IC_	Not quantitatively reported in the original study	Increased by 180% (ref. 45)
Nanofiber toughening (MWCNT 0.5 wt%)	DGEBF + DETD	Impact strength	35.56 kJ m^−2^ (ref. 46)	Baseline not reported in the same study; percentage cannot be calculated
Hyperbranched polymer (H30 10 wt%)	DGEBA + DETD + MeTHPA	Impact strength	Absolute value not directly comparable due to different testing configuration	Increased by 26.3% (ref. 47)
Hyperbranched polymer (H30 10 wt%)	DGEBA + DETD + MeTHPA	Tensile strength	115.6 MPa	Increased by 17.7% (from 98.2 MPa)^47^
Hyperbranched polymer (HPB 10 wt%)	DGEBA + MNA	Impact strength	Absolute value not directly comparable due to different testing configuration	Increased by 59.4% (ref. 17)
Hyperbranched polymer grafted boron nitride (HBP-BN 4 wt%)	DGEBA + MNA	Impact strength	Not quantitatively reported in the original study	Increased by 135% (ref. 48)
Hyperbranched polymer grafted boron nitride (HBP-BN 4 wt%)	DGEBA + MNA	Tensile strength	Not quantitatively reported in the original study	Increased by 72% (ref. 48)

a Because the cited studies employed different resin systems, specimen geometries, cryogenic testing methods, and evaluation criteria (*e.g.*, *K*_IC_, impact strength, tensile strength, and *G*_IC_), the values summarized here are intended primarily for qualitative and trend-based comparison rather than direct quantitative ranking. Relative improvements compared with the corresponding neat epoxy systems are reported wherever baseline data are available.

### 2.4 Thermal expansion mismatch and internal stress

Sections 2.1–2.3 detail the intrinsic molecular causes of cryogenic brittleness in neat polymers, while this section focuses on the coupled extrinsic aggravation mechanism in practical insulation systems: in composite insulation structures and electronic packaging of electrical equipment, insulating polymers are commonly combined with heterogeneous materials such as metallic conductors (*e.g.*, copper and aluminum), ceramic substrates (*e.g.*, alumina and silicon nitride), and fiber reinforcements (*e.g.*, glass fibers and carbon fibers), forming multiphase functional structures. In such composite systems, the mismatch in coefficients of thermal expansion (CTE) among different components is a major factor inducing internal stresses under extreme cryogenic conditions, thereby aggravating polymer embrittlement and degrading structural reliability.^31^ Its impact is often more significant than the intrinsic embrittlement behavior of a single polymer matrix.

The large differences in CTE between polymers and metals or ceramics constitute a key issue, as the CTE of polymers is typically one order of magnitude higher than that of metals and ceramics. When the composite system is cooled from room temperature or processing temperatures (typically 100–200 °C) to cryogenic temperatures such as 77 K or below, the shrinkage of each component becomes highly mismatched. Polymers tend to undergo much greater thermal contraction than heterogeneous materials, while interfacial bonding—including chemical bonding, mechanical interlocking, and van der Waals interactions—restricts this nonuniform deformation. As a result, significant thermal stresses are generated near the interfaces and propagate into the polymer matrix, forming complex three-dimensional internal stress fields.^32^

Under extreme cryogenic conditions, internal stresses induced by CTE mismatch act synergistically with molecular-scale mechanisms—including chain segment freezing, free volume contraction, and suppression of secondary relaxation—leading to intensified embrittlement. At this stage, the polymer remains deeply in the glassy state with very limited toughness and almost no plastic deformation capability. Internal stresses therefore cannot be effectively relaxed through segmental rearrangement or plastic flow and instead accumulate continuously in weak regions. These regions include interfacial defects (*e.g.*, voids and debonding sites), stress concentration points caused by filler agglomeration, microscopically nonuniform chain packing, and processing-induced defects.

When the accumulated internal stress exceeds the fracture strength of the polymer at cryogenic temperatures, rapid microcrack initiation and propagation occur, resulting in brittle fracture without plastic deformation.^33^ Microcracks at interfaces typically initiate along the bonding regions and extend into the polymer matrix, while microcracks within the matrix propagate rapidly under stress-driven conditions, simultaneously degrading the mechanical and dielectric properties of the composite insulation structure.

In practical service, electrical insulation components undergo repeated thermal cycling between cryogenic and room temperatures (*e.g.*, cooldown–warmup of superconducting magnets). Under cyclic conditions, CTE mismatch does not generate a static stress field but rather a fatigue-driving force. Each thermal cycle induces alternating tensile and compressive stresses at interfaces, promoting progressive microcrack growth. The crack growth rate per cycle (d*a*/d*N*) depends on the stress intensity factor range (Δ*K*) and the polymer's cryogenic fatigue resistance. Among toughening strategies, hyperbranched polymer modification and well-dispersed rigid nanoparticles (*e.g.*, nano-SiO_2_) have shown superior performance under cyclic cryogenic loading because they provide steady crack-tip blunting and deflection without undergoing phase separation or debonding that can accelerate under cyclic strain. Thermoplastic-toughened systems also perform well if the phase morphology remains stable, whereas rubber-toughened epoxies may experience accelerated fatigue damage if the rubber phase microcracks or dewets after multiple cycles.^33^

**Fig. 1:**
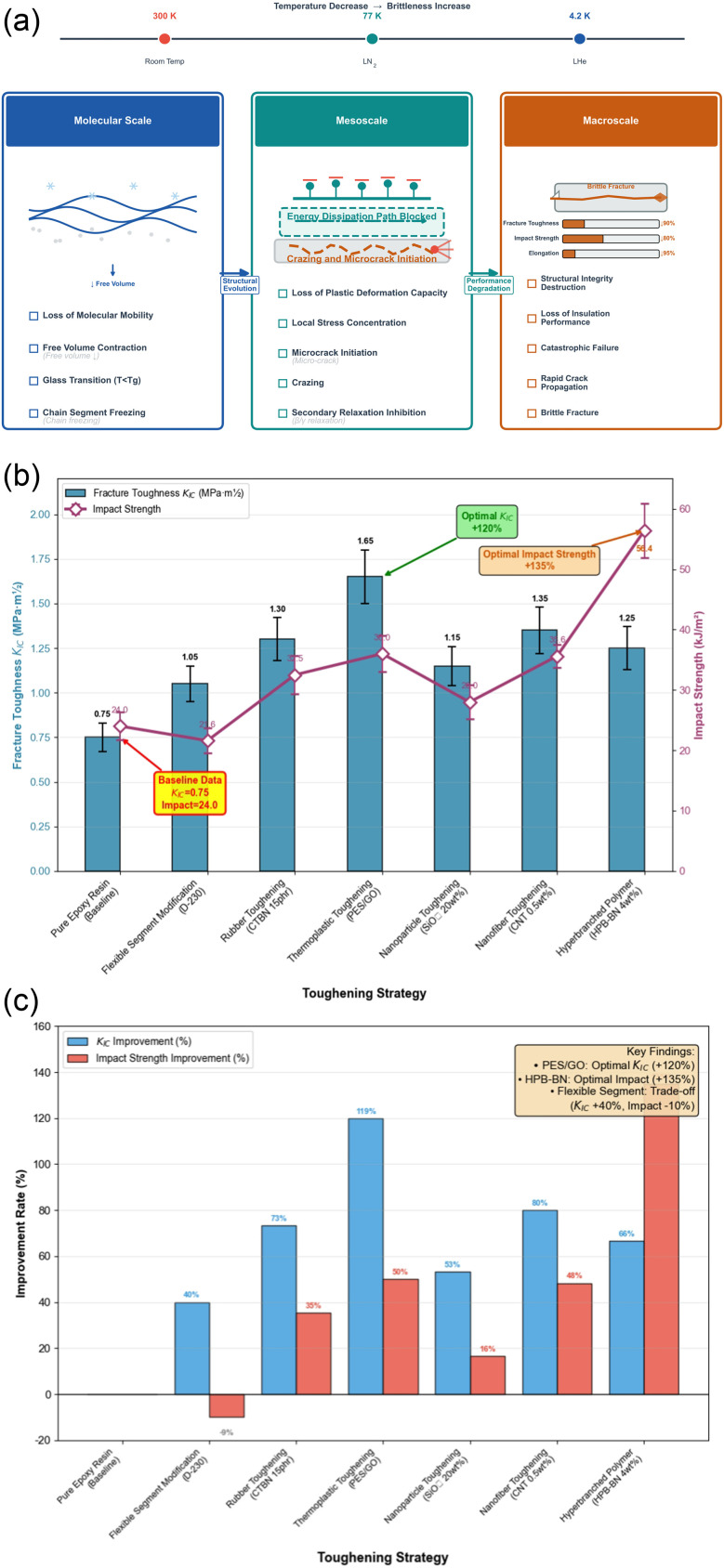
(a) Multi-scale correlation mechanism of polymer embrittlement in extreme cryogenic environment; (b) performance comparison of toughening strategies for electrical insulation polymers at cryogenic temperature for mechanical properties at 77 K; (c) performance improvement to pure epoxy at 77 K. Sources: ref. 1, 17, 31 and 32.

In summary, the embrittlement of electrical insulating polymers under extreme cryogenic conditions is a multiscale process involving the coupled effects of molecular chain freezing, free volume contraction, suppression of secondary relaxation, and macroscopic internal stress. The multiscale correlation mechanisms are schematically illustrated in Fig. 1(a).

## 3. Toughening strategies and material design at cryogenic temperatures

Cryogenic toughening strategies for insulating polymers can be broadly classified into intrinsic and extrinsic categories according to whether the toughening effect originates from modification of the polymer molecular network itself or from externally introduced phases/interfaces. Intrinsic toughening refers to molecular-level regulation strategies that directly alter chain mobility, crosslink topology, free volume, or secondary relaxation behavior within the polymer network, without relying on an independent second phase. In contrast, extrinsic toughening relies on the incorporation of additional phases such as elastomers, thermoplastics, nanoparticles, or nanofibers, where toughness enhancement is primarily achieved through mesoscale mechanisms including crack deflection, crack bridging, cavitation, particle debonding, and interfacial energy dissipation. Based on this criterion, the following discussion divides cryogenic toughening approaches into intrinsic molecular design and extrinsic composite reinforcement strategies.

### 3.1 Intrinsic toughening: molecular structure design

Intrinsic toughness design aims to suppress embrittlement at its molecular origin by regulating chain mobility, free volume, secondary relaxation, and network constraints. This category is especially suitable for insulation systems in which phase separation or conductive fillers must be avoided, because the toughening function is built into the polymer network rather than introduced as a separate phase.

#### 3.1.1 Flexible-segment incorporation targeting chain-segment freezing

The primary embrittlement mechanism addressed by flexible-segment design is the freezing of chain-segmental motion below *T*_g_. Introducing aliphatic, polyether, or siloxane segments into rigid epoxy or imide networks increases local rotational freedom and provides limited molecular mobility even at 77 K. For example, long-chain amine or anhydride curing agents can reduce the effective crosslink density and act as molecular springs that dissipate impact energy during crack initiation.^34–37^ The main mechanical benefit is improved crack-tip blunting and stress relaxation, but the electrical trade-off is that excessive flexible content may increase dipolar relaxation, dielectric loss, and thermal expansion, while also lowering *T*_g_ and dimensional stability. Therefore, flexible-segment design should be optimized as a low-dose mobility compensation strategy rather than as unrestricted softening.

#### 3.1.2 Crosslink-density and rigid-flexible network regulation targeting over-constrained glassy networks

A second intrinsic route is to moderate the crosslink density or construct a balanced rigid-flexible network. This strategy directly addresses the embrittlement caused by an over-constrained three-dimensional network, in which segmental rearrangement and secondary relaxation are severely restricted at cryogenic temperatures.^38–40^ Reducing crosslink density or introducing flexible junctions can increase fracture toughness by allowing localized deformation around crack tips. However, high crosslink density is often beneficial for dielectric strength, thermal resistance, solvent resistance, and long-term dimensional stability. The design trade-off is therefore between mechanical energy dissipation and electrical/thermal robustness: insufficient crosslinking may improve toughness but can reduce breakdown strength, raise dielectric loss, and increase creep or shrinkage during thermal cycling.

#### 3.1.3 Branched and hyperbranched architectures targeting free-volume collapse and internal stress accumulation

Hyperbranched and dendritic modifiers contain internal cavities and numerous terminal groups, which can introduce intramolecular free volume and topological constraints that create localized low-density regions and stress-buffering effects—even if partial infiltration by matrix chains occurs—thereby improving stress redistribution in densely packed cryogenic polymer networks.^41,42^ This strategy mainly counteracts free-volume contraction and internal residual-stress accumulation, two mechanisms that accelerate crack initiation under rapid cooling. Representative hyperbranched epoxy or polyester modifiers can reduce processing viscosity and create localized energy-dissipation zones without forming large soft domains. Electrically, their influence is often smaller than that of rubber particles, but polar terminal groups or unreacted functional groups may increase dielectric loss or moisture sensitivity. Thus, terminal-group chemistry and compatibility with the matrix should be controlled to preserve insulation stability while maintaining the desired free-volume effect.

#### 3.1.4 Secondary-relaxation regulation targeting the loss of microscopic energy-dissipation pathways

Because beta and gamma relaxations are key low-temperature energy-dissipation processes in glassy polymers, molecular units that remain locally mobile at cryogenic temperatures can delay brittle crack propagation.^21,26,27^ Examples include ether linkages, siloxane segments, flexible side groups, and controlled local heterogeneity that promote low-activation-energy motions. The mechanical advantage is improved impact resistance without relying on large-scale plastic flow. The electrical trade-off is that the same polar or mobile units may contribute to dielectric relaxation and loss, particularly under alternating electric fields. Consequently, secondary-relaxation design should prioritize low-polarizability flexible units and avoid excessive dipole density.

Table 2 summarizes these strategy-mechanism relationships and highlights why cryogenic toughening cannot be evaluated by fracture toughness alone; dielectric strength, dielectric loss, *T*_g_, thermal expansion, and processability must be considered simultaneously.

### 3.2 Extrinsic toughening: composite and nanotechnology approaches

Extrinsic reinforcement introduces elastomeric, thermoplastic, rigid inorganic, fibrous, or two-dimensional phases into the polymer matrix. Unlike intrinsic design, these approaches do not primarily restore molecular mobility; instead, they create mesoscale toughening mechanisms such as crack deflection, crack pinning, shear banding, particle cavitation, fiber bridging, and interfacial pull-out. Their effectiveness is strongly controlled by phase morphology, interfacial bonding, filler dispersion, and the dielectric compatibility of the added phase.

#### 3.2.1 Elastomer-particle toughening targeting crack-tip stress concentration and limited matrix yielding

Rubber particles such as CTBN, natural rubber, or core–shell rubber can initiate particle cavitation and promote shear yielding around crack tips, thereby dissipating energy in otherwise brittle epoxy matrices.^49^ Although the matrix remains globally glassy at cryogenic temperatures, nanoscale deformation zones may still be activated locally under extremely high triaxial stress near cavitated particles or crack-tip regions. This strategy is designed to mitigate the inability of the glassy matrix to blunt cracks after chain motion and secondary relaxation have been suppressed. However, at cryogenic temperatures, the rubber phase may itself vitrify and lose deformability unless an elastomer with an extremely low *T*_g_, such as silicone rubber, is used. Electrically, elastomer domains may increase dielectric constant and dielectric loss, reduce thermal stability, and introduce interfacial polarization. Therefore, elastomer toughening offers high mechanical efficiency but must be limited and well dispersed in high-voltage insulation applications.

#### 3.2.2 Thermoplastic-phase toughening targeting suppressed plastic deformation and rapid crack propagation

Thermoplastics such as PES, PEK-C, and PI can form micron-scale particles or continuous phase-separated networks within thermosetting matrices.^50^ These thermoplastic domains can undergo local plastic deformation, bridge cracks, and force propagating cracks to follow tortuous paths. The corresponding embrittlement mechanism is the loss of matrix plasticity and the rapid propagation of sharp cracks under cryogenic loading. Compared with elastomers, thermoplastics generally offer a better balance between toughness, modulus, and dielectric performance because many engineering thermoplastics maintain relatively high *T*_g_ and low dielectric loss. The trade-off lies mainly in processing complexity, viscosity increase, and the need to control phase separation so that the domains toughen the matrix without forming defects or weak interfaces.

#### 3.2.3 Rigid nanoparticle toughening targeting free-volume collapse, crack-path instability, and interfacial stress concentration

Rigid nanoparticles such as nano-SiO_2_ and nano-Al_2_O_3_ can deflect, pin, and branch cracks, while strong particle–matrix interfaces can induce localized interfacial rearrangement and crack-tip blunting within confined nanoscale regions.^51^ This strategy addresses the lack of energy-dissipating crack paths in cryogenic glassy polymers and can also reduce local stress concentration when particles are uniformly dispersed. In electrical insulation systems, well-dispersed inorganic nanoparticles may improve dielectric strength by suppressing charge transport and homogenizing the microstructure. Conversely, agglomerates, voids, or poorly bonded interfaces can become both mechanical crack-initiation sites and electrical field-enhancement sites. Thus, the mechanical–electrical balance depends less on particle loading alone and more on surface functionalization, dispersion quality, and interface integrity.

#### 3.2.4 Nanofiber and two-dimensional filler reinforcement targeting thermal-stress accumulation and unstable crack growth

Carbon nanotubes, graphene, and related one- or two-dimensional fillers can bridge cracks, undergo pull-out, and increase the tortuosity of crack propagation. Their high thermal conductivity can also reduce local thermal gradients and thermal-stress accumulation during cooling, indirectly mitigating CTE-mismatch-induced cracking. However, because CNTs and graphene are electrically conductive, the same continuous network that improves thermal transport may create leakage paths, reduce dielectric strength, or generate local field concentration. For electrical insulating polymers, the key design principle is to remain below the electrical percolation threshold, use insulating surface coatings or hybrid fillers when necessary, and prioritize dispersion architectures that improve crack resistance without sacrificing insulation safety.

The above classification indicates that intrinsic strategies are generally preferable when dielectric purity, homogeneous insulation behavior, and processing simplicity are dominant requirements, whereas extrinsic reinforcement is more suitable when high fracture toughness, thermal-stress management, or structural load bearing is required. In cryogenic electrical equipment, the optimal formulation is often a hybrid design: intrinsic molecular mobility is used to delay crack initiation, while carefully selected second phases are used to arrest crack propagation.

### 3.3 Trade-off-guided design for coupled electrical and mechanical performance

A practical cryogenic insulating material should not be selected solely according to *K*_IC_ or impact strength. Toughening mechanisms that increase molecular mobility or introduce interfacial polarization may improve mechanical reliability while reducing dielectric strength or increasing dielectric loss. Conversely, strategies that increase rigidity, filler content, or interfacial bonding may improve dielectric strength and modulus but can intensify thermal-expansion mismatch or stress concentration. Therefore, each toughening route should be evaluated by a coupled performance window that includes fracture toughness, impact strength, *T*_g_, dielectric strength, dielectric loss, CTE, thermal conductivity, and stability under thermal cycling.

For flexible-segment and low-crosslink designs, the central balance is mobility *versus* dielectric/thermal stability. For elastomer and core–shell rubber systems, the balance is cavitation-induced toughness *versus* interfacial polarization and cryogenic vitrification of the soft phase.^51^ For thermoplastic toughening, the balance is phase-separated plastic deformation *versus* processing viscosity and morphology control.^52^ For rigid nanoparticles, the balance is crack deflection and dielectric reinforcement *versus* agglomeration-induced defects. For CNTs and graphene, the balance is bridging and thermal-stress relief *versus* electrical percolation risk. These trade-off mechanisms should be explicitly reported together with cryogenic mechanical data so that the suitability of a strategy for high-voltage cryogenic insulation can be judged more accurately.

To provide a quantitative reference for such trade-off evaluations, Table 3 summarizes the comprehensive properties of modified epoxy resin systems at 77 K, including fracture toughness (*K*_IC_), impact strength, glass transition temperature (*T*_g_), and dielectric strength. Because the data summarized in Table 3 originate from different literature sources employing distinct resin formulations, testing standards, and cryogenic characterization methods, the comparison should be interpreted as a mechanism-oriented and trend-based evaluation rather than a strict one-to-one quantitative ranking. Fig. 1(b) and (c) further present a visual comparison of the cryogenic mechanical performance of modified epoxy systems. Specifically, Fig. 1(b) illustrates the distribution of fracture toughness and impact strength across different toughening approaches, while Fig. 1(c) shows the relative improvement compared with neat epoxy. Together, this quantitative summary demonstrates that no single strategy is universally optimal; instead, the selection of a toughening strategy must consider both the specific cryogenic embrittlement mechanism being addressed and the electrical performance requirements of the target application.

## 4. Challenges and perspectives

In the future research and development of electrical insulating materials for extreme cryogenic applications, it is imperative to transition from macroscopic performance descriptions to highly operational technical pathways to address increasingly severe environmental challenges. First, it is necessary to break through the current research limitation of focusing solely on single cryogenic factors and commit to building an *in situ* multi-physics observation platform capable of simultaneously simulating ultra-low temperatures, strong electromagnetic fields, continuous mechanical stress, and high-energy radiation environments. This will allow for the quantitative revelation of the microscopic fatigue and evolution mechanisms of polymer molecular chains under electro-mechanical–thermal coupling, providing a scientific basis for establishing more accurate material life prediction models.^53,54^

In addition, multi-scale simulation methods are expected to become essential tools for revealing cryogenic embrittlement mechanisms and guiding material design. Molecular dynamics (MD) simulations can be used to analyze chain segment freezing, free-volume evolution, secondary relaxation behavior, and interfacial molecular interactions at cryogenic temperatures. Coarse-grained and mesoscale models can further bridge nanoscale molecular mobility with microstructural heterogeneity, including phase separation, nanoparticle dispersion, and interfacial stress localization. At the macroscopic level, finite-element analysis (FEA) and phase-field fracture simulations can quantitatively predict thermal-stress accumulation, crack initiation, crack propagation, and thermo-mechanical coupling reliability under cyclic cryogenic loading. Future research should therefore establish integrated simulation-experiment frameworks capable of linking molecular structure, processing conditions, and multi-field service performance, which enables predictive and mechanism-guided design of next-generation cryogenic insulating polymers. Machine-learning-assisted materials informatics and data-driven surrogate models may further accelerate the discovery and optimization of cryogenic insulation systems with balanced electrical and mechanical performance.

To address the performance trade-offs where toughening modifications often lead to increased dielectric loss, decreased thermal stability, or a surge in processing viscosity,^30,55^ future research should shift toward developing intrinsically low-loss flexible curing systems. Examples include the introduction of perfluoropolyether or polysiloxane long chains with low polarity and high free volume characteristics, alongside the exploration of low-filler-content nanocomposite technologies based on magnetic field-induced orientation. This approach aims to achieve the synergistic optimization of cryogenic toughness and dielectric strength through the directional distribution of interfacial stress without compromising processing feasibility.^1,56^

Furthermore, considering that molecular segment movement is highly frozen at extreme cryogenic temperatures, research focus should be placed on artificially constructing “molecular-level cavities” within the cross-linked network *via* hyperbranched structures to provide stress buffering.

Additionally, developing intelligent self-healing systems that can be triggered by local discharge heat or assisted by shape memory effects to achieve autonomous closure and healing of microcracks will become a visionary direction for solving cryogenic embrittlement failure at its source and enhancing the system reliability of superconducting electrical equipment.

## 5. Conclusions

Extreme cryogenic environments impose significant constraints on the toughness of electrical insulating polymers. The primary origin of cryogenic embrittlement lies in the freezing of molecular chain segmental motion and the reduction of effective molecular mobility associated with free volume evolution.^56^ Strategies such as interfacial anchoring to regulate crosslink density and tailoring the activation temperature of secondary relaxation processes can help alleviate segmental freezing and improve energy dissipation capacity.^57^

Effective improvement of cryogenic crack resistance requires a multiscale synergistic approach spanning molecular structure design to macroscopic composite modification. Representative strategies include the incorporation of flexible chain segments, regulation of network architecture, hybridization with elastomeric or thermoplastic particles, and the application of nanotechnology to enhance interfacial interactions. For instance, polydopamine nanoparticles have been reported to enhance the fracture toughness of epoxy at −196 °C by up to 610%, which shows the effectiveness of multiscale synergistic toughening.^58^

Future research should place greater emphasis on the coupled effects of multiple extreme fields and pursue comprehensive optimization of mechanical, electrical, and thermal properties. Integrating computational simulation with experimental design will enable on-demand material development and precise fabrication, thereby providing highly reliable insulating materials for next-generation technologies such as superconducting systems and deep-space exploration.

## Author contributions

The authors confirm their contributions to the paper as follows: conceptualization, Yuhan Deng and Wanchuan Liu; methodology, Wanchuan Liu and Yuhong Yuan; software, Mengjin Li; validation, Mengjin Li; formal analysis, Jingrui Liu; investigation, Jingrui Liu and Baowen Xing; resources, Baowen Xing; data curation, Mengjin Li; writing—original draft preparation, Mengjin Li and Jingrui Liu; writing—review and editing, Yuhong Yuan and Jingrui Liu; visualization, Yuhong Yuan and Mengjin Li; supervision, Yuhan Deng; project administration, Jingrui Liu. All authors reviewed the results and approved the final version of the manuscript.

## Conflicts of interest

The authors declare no conflicts of interest to report regarding the present study.

## Data Availability

The data that support the findings of this study are available from the corresponding author, Jingrui Liu, upon reasonable request.
